# Tensor based multichannel reconstruction for breast tumours identification from DCE-MRIs

**DOI:** 10.1371/journal.pone.0172111

**Published:** 2017-03-10

**Authors:** X. -X. Yin, S. Hadjiloucas, J. -H. Chen, Y. Zhang, J. -L. Wu, M. -Y. Su

**Affiliations:** 1 Centre for Applied Informatics School of Engineering and Science, Victoria University, Melbourne, Australia; 2 School of Systems Engineering and Department of Bioengineering, University of Reading, Reading RG6 6AY, United Kingdom; 3 Tu & Yuen Center for Functional Onco-Imaging, Department of Radiological Sciences, University of California, Irvine, CA, United States of America; 4 Department of Radiology, EDa Hospital and I-Shou University, Kaohsiung, Taiwan; 5 School of Computer Science, Fudan University, China; 6 Department of Radiology, Affiliated Zhongshan Hospital of Dalian University, Dalian, Liaoning, China; Beijing University of Technology, CHINA

## Abstract

A new methodology based on tensor algebra that uses a higher order singular value decomposition to perform three-dimensional voxel reconstruction from a series of temporal images obtained using dynamic contrast-enhanced magnetic resonance imaging (DCE-MRI) is proposed. Principal component analysis (PCA) is used to robustly extract the spatial and temporal image features and simultaneously de-noise the datasets. Tumour segmentation on enhanced scaled (ES) images performed using a fuzzy C-means (FCM) cluster algorithm is compared with that achieved using the proposed tensorial framework. The proposed algorithm explores the correlations between spatial and temporal features in the tumours. The multi-channel reconstruction enables improved breast tumour identification through enhanced de-noising and improved intensity consistency. The reconstructed tumours have clear and continuous boundaries; furthermore the reconstruction shows better voxel clustering in tumour regions of interest. A more homogenous intensity distribution is also observed, enabling improved image contrast between tumours and background, especially in places where fatty tissue is imaged. The fidelity of reconstruction is further evaluated on the basis of five new qualitative metrics. Results confirm the superiority of the tensorial approach. The proposed reconstruction metrics should also find future applications in the assessment of other reconstruction algorithms.

## Introduction

Currently breast cancer is listed as the second most common cause of deaths for women [1]. Over 1.3 million women worldwide that undergo tumour screening are diagnosed with breast cancer each year, making it one of the most common forms of cancer. Traditional two-dimensional digital mammography is being supplanted by three-dimensional digital breast tomosynthesis (DBT), and contrast-enhanced digital mammography (CEDM) is a test that images vascularity as well as anatomic abnormalities. Screening breast ultrasound is an increasingly requested supplemental screening technique in women of all breast densities [2]. Although mammography is regarded as the gold standard for the diagnosis of breast tumor, and ultrasound is also commonly used, recently breast MRI has also been gaining ground as an alternative modality in clinical practice [2–4]. A reason for potentially considering alternative modalities has been the documented evidence that false negative errors are elevated when targets are rare (low prevalence cases) [5] as well as the potential harmful effects associated with repeated examinations [6]. In addition, a new more informative modality called dynamic contrast enhanced MRI, (DCE-MRI) has been recently introduced, this makes use of a contrast enhancement agent to improve on the retrieval of 3D spatial information of lesions as well as provide temporal information on lesion physiology (showing variations in contrast agent uptake rates), allowing for more accurate assessment of lesion extent and better lesion characterisation [7]. Currently there is good agreement between mammographic measures of volumetric breast density to MRI results [8, 9]. Results from a systematic review and meta-analysis of several peer-reviewed studies in PubMed applying dynamic contrast-enhanced breast MRI as an adjunct to conventional imaging (mammography, ultrasound) [10], have shown that indeed breast MRI demonstrates excellent diagnostic performance in cases of non-calcified equivocal breast findings detected through conventional imaging. A drawback identified through that study was that there are cases where there is substantial heterogeneity in imaged tissues which show a prevalence of malignancy, so the criteria for correctly discriminating between different lesions would need to be better defined. For example, the increased vascular permeability associated with angiogenic processes leads to a wider variety of tissue types that need to be identified [11].

As a result of the above studies, a need for better processing of DCE-MRI datasets clearly emerges, which once successfully addressed it could eliminate the current bottleneck to the wider proliferation of the technique in a clinical setting. It is indeed the case that in order to address the above shortcomings, the aim of such processing should be to identify methods that would reliably reconstruct tumour segments while at the same time provide an interpretation of imaged lesions and an assessment of disease proliferation. The automatic detection of specific features in malignant tumours from spatiotemporal data addressed in the current contribution aims to address this issue. Furthermore, the contribution is of relevance across several emergent interdisciplinary topics within the computer science and biomedical imaging communities where spatiotemporal datasets might have been obtained from a variety of transduction technologies or alternative imaging modalities [12]. It is indeed the case that there is still a shortage of solutions that can reliably extract features from such multi-dimensional datasets and in order to successfully address current shortcomings, significant innovation is required from an algorithmic perspective [13].

Image processing techniques can be used to extract quantitative information on lesion morphology, volume and kinetics, as well as to distinguish viable from nonviable tissue [7]. In dynamic pattern recognition methods, the emphasis has been on either high temporal resolution and empirical analyses [7, 14–17] or on high spatial resolution with a stand-alone morphologic feature extraction [7, 18–22]. Even though time-series analysis enable radiologists to infer information regarding the tissue state, such assessment is a time-consuming task, because of spatiotemporal lesion variability. Currently, most studies consider aggregate measurements for tumour morphological characterization [7, 19, 20] with an initially model-free [19, 20] and data-driven [23, 24] segmentation according to manually marked region of interest (ROI). In addition, the analysis of four-dimensional DCE-MRI data with correlation to multi-parametric data from other MRI imaging sequences forms an impediment to interpreting DCE-MRIs for screening of breast tumours [25].

In order to help overcome these limitations, considerable efforts are currently being made on the development of computer-aided diagnosis (CAD) algorithms [26]. In clinical practice, an automated kinetic assessment protocol may be implemented to colour-code the intensity changes per voxel to enable the further interpretation of patterns resulting from contrast enhancement (persistent, plateau and washout enhancement) across a series of MRI volumes [27], but the technique is not fully automated and requires continuous feedback from experts. A further major challenge in the diagnosis of breast DCE-MRI is the spatiotemporal association of tumour enhancement patterns, a task that humans are not as optimized to perform. This is so because the morphological pattern of a tumor in DCE-MRIs dynamically change due to the diffusion of the contrast-enhanced agent which modifies disproportionally the signal enhancement factor of local voxels. Likewise, the kinetic patterns of enhancement may be different across various parts within a tumor [7]. Although conventional computer-aided detection (CAD) systems can facilitate the marking of the most suspicious locations for tumours in breast tissue, thus assisting radiologists on the analysis and interpretation of DCE-MRIs, there is still some risk of misinterpreting or overlooking breast lesions [28, 29], and inter- and intra-observer variability even by experts can be encountered [30].

Common practice in these methods is to process the imaged 3D volumes separately, and then incorporate the temporal information into the spatial databases through a separate processing step. Only a few authors have presented algorithms that explore the spatiotemporal association of tumour enhancement patterns using computer-aided diagnosis [21, 25, 31, 32]. The method developed by Zheng et al. in 2009 [21] combines features in both the time and spatial domains to define a spatiotemporal enhancement pattern. Such practice can lead to an improved characterization of breast tumours. The method relies on the Fourier transformation and pharmacokinetic modelling of the datasets to extract features associated with the various temporal enhancements and the calculation of moment invariants and Gabor texture features for refining the coarse segmentations of tumours manually. That method, however, is not fully automated, since initial manual segmentation by experts is performed. Gubern-Merida et al. in 2015 [25] developed a multi-stage approach that uses ‘blob’ features in combination with kinetic and morphological information of lesions mapped using motion-corrected data. However, their method still requires the manual selection of appropriate seed locations for lesion segmentation. In addition, such approach can be subjective, relying on interpretation by experts on site. Because of inconsistencies due to textural differences, less accurate breast segmentation may result. An alternative approach is feature extraction through image reduction. This enables tumour identification on the basis of the dominant features present in the image. For example, in [31], principal component analysis (PCA) is conducted on enhanced and scaled datasets for an entire object region obtained by DCE-MRI. A drawback of traditional PCA is that it is only well suited for two-dimensional MRI image analysis, and cannot account for spatiotemporal changes from images acquired at different time instances. Finally, automatic selection of extracted spatiotemporal features is another technique that can lead to improved detection. In [32], for example, a fast orthogonal search algorithm that uses QR decomposition is proposed to automatically select orthogonal feature vectors with the most predictive value from a large pool of potential features. A drawback in that approach is that it requires the calculation of a large number of features and therefore increases computational load when dealing with multiple voxels obtained at fine resolution. Most experts accept that future feature extraction techniques should consider the associations between spatial and temporal features of high-dimensional images, and that there is a need to design algorithms for effective multi-dimensional decomposition, feature extraction and segmentation/classification. This paper, addresses this issue by proposing a novel hybrid data transform and classification approach that uses spatial and temporal feature extraction techniques by proposing a tensor based multichannel reconstruction algorithm. The algorithm is then used for feature extraction and classification of imaged tumours from healthy tissues. To achieve this objective, in the current study, several post-contrast image datasets covering the whole breast were acquired and analysed at discrete time stamps.

The paper is organised as follows: the first part describes the DCE-MRI data acquisition modality and the associated multi-dimensional data structures obtained through a sequence of images. The methodology section describes the multi-channel reconstruction methodology. Temporal PCA analysis is applied on factorized tensors after taking into consideration the spatio-temporal alignment of DCE-MRIs. Methods to select the effective number of voxels for accurate multi-channel reconstruction are discussed in a subsection that focuses on dataset pre-processing; the aim of these routines is to improve on the signal to noise ratio per reconstructed voxel. The validation section firstly illustrates the resulting images after the application of pre-processing operations on original DCE-MRIs. Then, several quality metrics are defined to validate the resultant reconstruction. In order to further evaluate the advantages of the proposed technique, we examine the ability of the multi-channel reconstruction to (i) remove heterogeneous intensity distribution in the detected tumour region; (ii) to effectively suppress imaged voxels of fatty tissue from background; and (iii) to achieve uniformly enhanced intensity distributions with increased image contrast between tumours and background; finally (iv) we also discuss the limitation of the current reconstruction algorithm and analyse its image reconstruction ability in relation to several pre-defined qualitative matrices. The quality of reconstructed tumour images is also compared with enhanced scaled (ES) images obtained using traditional FCM which focuses on spatial intensity distribution of DCE-MR scans. The fidelity of reconstruction using the proposed hybrid algorithm is discussed on the basis of the qualitative metrics proposed. Finally, some concluding remarks are also provided.

## Subject treatment and MR imaging protocol

**Fig 1:**
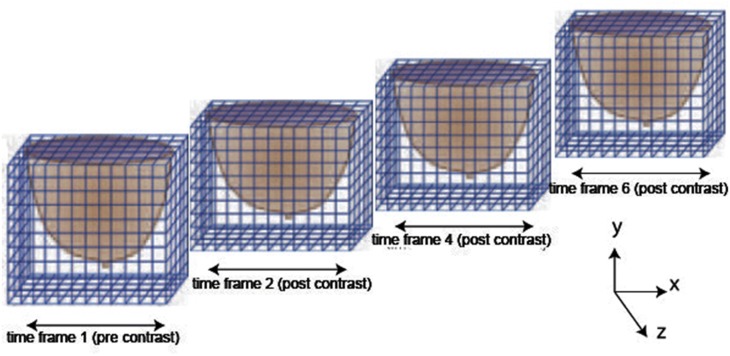
Cartesian segmentation of dynamic contrast enhanced MR image regions acquired at different time frames used for feature extraction in the current study, after [33].

Image reconstructions of breast tumours on eleven different representative patient cases are conducted on the basis of their recorded DCE-MRIs. Additional independent assessment and classification of lesion types (mass-like or non-mass-like) as well as assessment of change in size or metastatic tendency from different imaging sessions is performed by an experienced radiologist, with the radiological reports providing an independent assessment of classifier performance. The DCE-MRI datasets are grouped into the following seven categories: benign lesions with predominantly fibrocystic changes, fibroadenomas, tubular adenomas, granular cell tumours, in situ ductal carcinomas, invasive ductal carcinomas, and finally mucinous carcinomas. The DCE-MRI dataset used in the current image analysis consists of one baseline 3D MR reference image before contrast agent injection, (this is associated to the first time frame); followed by six 3D post-contrast images obtained at subsequent time frames. Time frames are obtained successively at 60 second intervals. In addition, volume MR images from different sessions are also studied to assess disease proliferation. Each volume MR image consists of multiple two-dimensional image layers. Each volume image consists of several intensity values corresponding to voxels in a Cartesian grid. Each image is composed of 448 × 288 × 160 voxels in *x*, *y* and *z* axis directions, respectively. In the current study, we are only concerned with one side of the breast where there are tumour regions of interest as illustrated in Fig 1.

The breast MR images were acquired with a 3.0 Tesla MR scanner (Magneton Skyra, Siemens Medical Solutions, Erlangen, Germany). The 16-channel imaging receiver configuration of the sentinelle breast coil consisted of two lateral 4-channel coil elements and an 8-channel middle element coil. The MRI protocol consisted of a high spatial resolution setting, with non-contrast-enhanced 2D fast spin echo (FSE) T1WI in axial sections followed by a dynamic contrast-enhanced MR imaging (DCE-MRI) with FSE sequences. The imaging parameters for DCE-MRI were: TR/TE = 4.36/1.58 ms, a flip angle of 10 degrees, a matrix size = 384 × 288, with the number of signal averages set to 1, a field of view of 30 cm, and a slice thickness of 1.0 mm. In total one pre-contrast imaging frame and six post-contrast imaging frames were acquired.

## Methodology

The proposed analysis is based on a novel dynamic tensor reconstruction algorithm [34] aimed to achieve spatio-temporal feature extraction through principal component separation. A higher-order singular value decomposition (HOSVD) is used for tensor factorization of MRIs at each time frame, in order to decompose dynamic (temporal) 3D MRIs to three different modes of dynamic (temporal) 2D basis images. In order to extract the dominant component of temporal variation, principal component analysis (PCA) on one of the temporal sets of 2D basis images was performed. The PCA procedure is repeated for each of the three different modes (spatial orientations). For the final volume image reconstruction, tensor synthesis is performed by linearly combining the features extracted through PCA. An FCM analysis is conducted to assess the fidelity of identify the reconstructed tumours ensuring maximum visual separability of image features.

The proposed high dimensional image data transformation technique enables the analysis of spatio-temporal features and allows for signal from fatty tissue to be attenuated reducing the influence of the background to regions of interest. The approach leads to improved fidelity; furthermore it provides additional consistency and tumorous feature enhancement in the reconstructed images.

## Ethics statement

Human studies were approved by Victoria University Committee and by the Institutional Review Board. MR imaging was conducted in accordance with guidelines defined by Affiliated Zhongshan Hospital of Dalian University to achieve safe and reliable scanning. The experiment was approved specifically by the ethics committee. Written consent was obtained from each case subject after the imaging procedures had been conveyed.

### Tensor reconstruction incorporating PCA

Tensors enable multilinear mappings over a set of vector spaces. Under a tensorial framework, the four-dimensional objects represented using DCE-MRIs are treated as a fourth order tensor, and geometric shape objects associated with the 3D spatial image dataset are treated as a third order tensor. A third order tensor has a directional definition in space so any associated spatial matrix consists of three directional slices: horizontal, vertical and frontal.

Tensor factorisation of a 3D spatial matrix is a universal methodology that is well suited to the analysis of an ensemble of volume images. The tensor decomposition method adopted in the current study is based on the standard Tucker decomposition [35–37]. The associated terminology adopted was coined by P. Kroonenberg in the 1980s [38], and is also referred to as multilinear SVD or HOSVD (higher-order SVD) after L. De Lathauwer [39]. The advantage of the HOSVD approach is that it allows estimation of the dimension of the core tensor by analysing the distribution of singular values [40].

Before conduct the tensor reconstruction, the intensity-scaled (IS) dynamic datasets are loaded into MatLab (v. R2013b, MathWorks, Natick, MA) and their corresponding enhancement-scaled (ES) datasets are generated. Enhancement of the ES data is defined as the difference per voxel in the intensity of the post-contrast and pre-contrast images. In ES datasets, the reconstruction is performed on region of interest (ROI) through the use of a pre-processing step according to morphological operations and standard FCM methods. A dynamic tensor data structure is introduced to store the DCE-MR image datasets, as this provides a simple way of extracting data from different dimensions. Another advantage of adopting a tensorial framework in our data structure is that the DCE-MR image data can be easily projected in different directions by using tensor or kronecker products. Tensor factorization is conducted on each three-dimensional (3D) MRI image by decomposing it into three two-dimensional (2D) subspaces (basis images) that are, respectively, associated with each mode (spatial orientations) of observations. These three-modes of dynamic basis images are further aligned to different time frames. For added clarity, we call these aligned basis images with time course as a temporal set of basis images at a different mode.

With the use of HOSVD, the dynamic ES dataset (a dynamic tensor $X_{\tau }$) is decomposed into three-mode basis image matrices $A_{\tau }^{\iota }$ and a core tensor *C*_*τ*_, where *ι* = 1, 2, 3 is associated with each mode of basis images; *τ* = 1, 2, …, 6 corresponds to a single time frame. PCA is applied on a temporal set of basis images. The temporal signal intensity variations $v_{i}^{\tau \iota }$ for each pixel within the decomposed basis image at each mode are associated with a state vector: $u_{i}^{\iota }=u_{i}^{1\iota },u_{i}^{2\iota },\ldots ,u_{i}^{n\iota }$ (*n* = 6 for ES datasets). The set of all state vectors in one mode of the basis images over a pre-determined time course is defined as ${ϒ}^{\iota }=\left\{u_{i}^{\iota }\right\},1\leq i\leq \epsilon$ with *ϵ* the number of pixels in the basis image at a different mode *ι*. The first-order covariance matrix of ϒ^*ι*^, Δ^*ι*^, is calculated according to:

$$\Delta^{\iota }=\frac{1}{\epsilon }\sum_{u_{i}^{\iota }\in {ϒ}^{\iota }}\left(u_{i}^{\iota }-{\bar{u}}^{\iota }\right){\left(u_{i}^{\iota }-{\bar{u}}^{\iota }\right)}^{T}\;\text{and}\;{\bar{u}}^{\iota }=\frac{1}{\epsilon }\sum_{u_{i}^{\iota }\in {ϒ}^{\iota }}u_{i}^{\iota }$$ (1)

**Fig 2:**
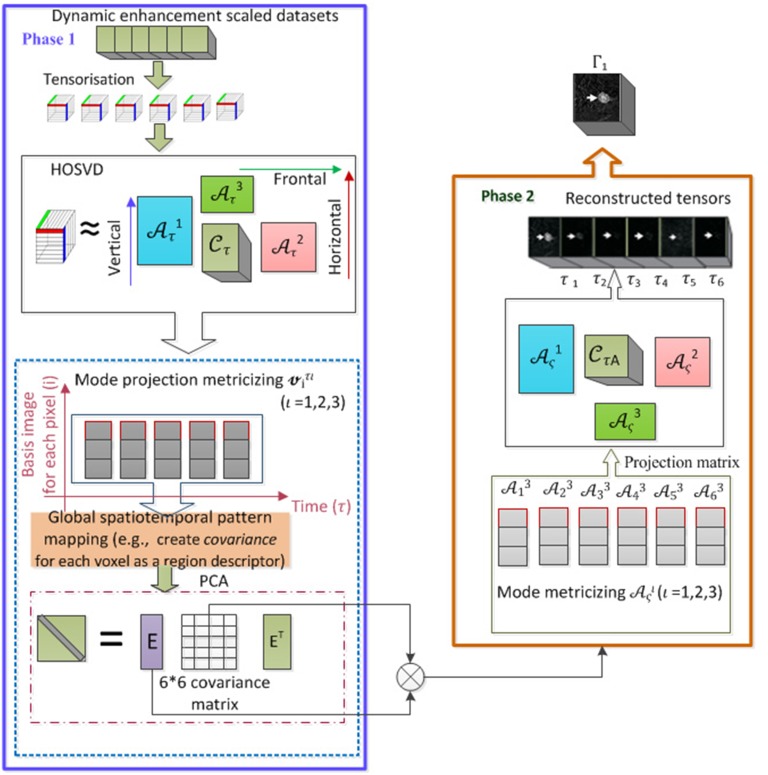
Illustrates the proposed multi-channel tensor reconstruction algorithm.

A linear PCA transformation is then applied to obtain the corresponding eigenvectors $E_{\varsigma }^{\iota }=\left\{e_{\varsigma }^{\iota }\right\}$, and eigenvalues λ = {λ_1_, λ_2_, …, λ_6_} by solving λ**E** = Δ**E**. A PCA of dynamic basis image datasets at each of the image modes yields 6 eigenvectors. After indexed and sorted according to their eigenvalues, the eigenvector corresponding to the largest eigenvalue is called first channel eigenvector, and so on. As a result, a new mode vector is re-constructed $A_{\varsigma }^{\iota }=\Delta^{\iota }E_{\varsigma }^{\iota }$ for each of the different channels (state points) (*ς* = 1, 2, …, 6). We matricise **A** to $A\in N^{\iota }\times M^{\iota }$, to generate *ι* modes of basis images. In order to reconstruct a tensor for a 3D MRI approximation, we calculate the tensor product between the averaged core tensor and three modes of filtered basis images. The resultant reconstruction based on the first channel eigenvector retrieves well the spatial structure of tumours with uniform enhancement in intensity so subsequent eigenvector values are filtered out. That is $\Gamma_{\varsigma }=C_{\tau A}\;\times_{1}\;A_{\varsigma }^{1}\;\times_{2}\;A_{\varsigma }^{2}\;\times_{3}\;A_{\varsigma }^{3}$, where $C_{\tau_{A}}=\frac{1}{3}\sum_{\tau =1}^{3}C_{\tau }$, and *ς* = 1. Finally, we reconstruct the spatio-temporal features in a 3D space. Tensor based multi-channel reconstruction models successfully preserve the intrinsic structures in an image providing a higher contrast per voxel. The generated images, therefore, convey improved diagnostic information. The procedure also allows the multi-channel reconstruction of spatial and temporal features simultaneously in relation to DCE-MRIs under a uniform tensor framework. Fig 2 illustrates the flow chart of this proposed multi-channel reconstruction algorithm.

**Fig 3:**
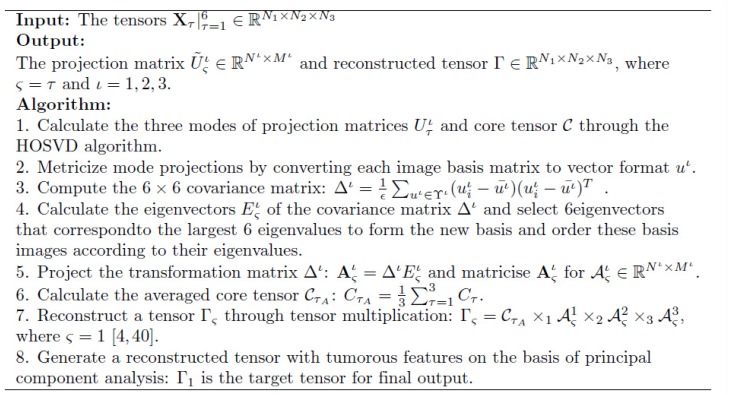
Pseudo code of tensor reconstruction.

The pseudo code for multi-channel tensor reconstruction is illustrated in Fig 3. Finally, the multi-channel reconstruction incorporates the FCM technique to segment the tumour region effectively.

### Baseline normalization pre-processing

**Fig 4:**
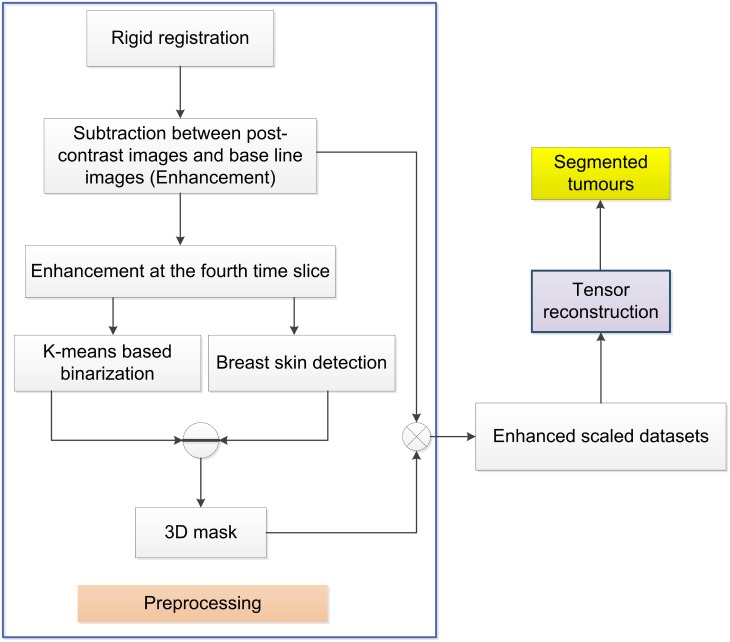
Illustrates the entire procedure for the segmentation of reconstructed tumour images, including both the pre-processing as well as the associated tensor reconstruction operations.

A necessary pre-processing step in the above reconstruction is the calculation of a mask that will enable the selection of the number of voxels that will ensure an accurate multi-channel reconstruction. For example, in the case where one considers that the fourth post contrast enhanced image shows the largest enhancement, in order to obtain the 3D image masks, firstly, we subtract the pre-treatment baseline image from this fourth post contrast image. The resultant volume image shows tumours that have been enhanced after contrast agent injection compared to the base line image. Secondly, the tumour regions that show obvious enhancement are approximately identified using the FCM based method. In order to avoid missing tumour voxels, the third step is to adopt a morphological dilation operation to generate a 3D image mask. Finally, each of the enhancements (subtraction between post contrast images and a baseline image) convolves with the 3D image mask for further reconstruction of the image time series. Before the start of the image processing, rigid transforms are conducted to ensure the alignments between pre-contrast enhanced MR image and post-contrast enhanced MR images. Fig 4 illustrates the entire procedure for the segmentation of reconstructed tumour images.

There are three advantages from this pre-processing operation. Firstly, there are no tumorous tissue voxels missed while at the same time noisy voxels associated with a large intensity background are filtered out. The traditional FCM algorithm efficiently identifies voxels displaying higher intensity as being related to tumorous regions. Through the use of morphological dilation operations, we enlarge the regions where there are suspected tumours, these have reduced intensity compared to the enhanced tumour patterns. Secondly, there is a reduction in computational complexity due to reduced amount of voxels that need to be processed. Finally, the pre-process step makes the number of voxels in the spatial domain comparable with the number of voxels in the time domain, so as that the multi-channel reconstruction procedure can be effectively conducted.

## Results and discussion

The algorithm has been implemented in MATLAB version R2013a on a personal computer running Windows 7 with an Intel(R) Core(TM) i5-3470 CPU (3.20 GHz) and 8 GB of memory. On this platform, it takes about 3.6972 seconds to process one case of breast DCE-MRI to complete the reconstruction. Considering that these results are obtained with MATLAB on a standard PC, the processing times are fast, and our method can be incorporated into most assisted-diagnosis systems providing a result within a very short time frame from a patient perspective, especially when compared to a manual evaluation of tumour position.

We combine FCM analysis after performing the proposed reconstruction with the segmentation process of the imaged tumours. The resultant segments are compared with those reconstructed using conventional FCM techniques.

### Pre-processing

**Fig 5:**
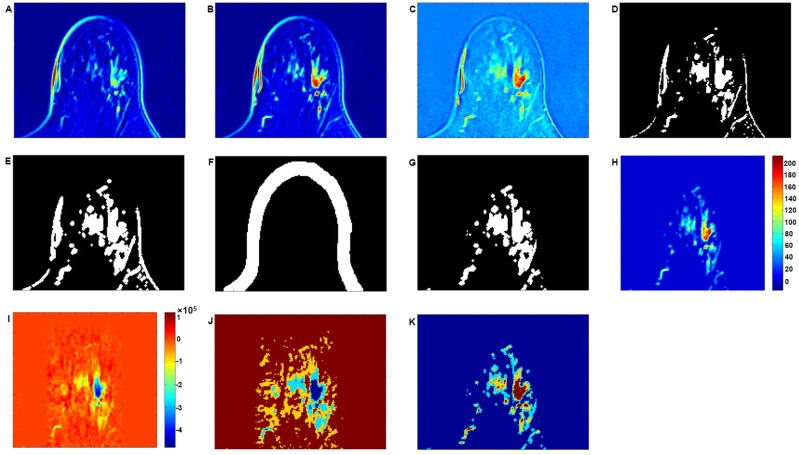
Illustration of the procedure in relation to multi-channel image reconstruction after the application of pre-processing operations on the original DCE-MRIs. (A) Illustration of a single layer associated with the image baseline before the contrast enhanced agent is injected. (B) Post contrast enhanced MRI at the fourth time frame for the same layer. (C) Result from the subtraction of (A) from (B). (D) FCM clustering for initial binarization. (E) Application of morphological dilation operation on the image of (D); (F) Extraction of imaged breast skin tissues. (G) Subtraction of (F) from (E) to achieve a binarization image that is used to find voxels that are most related to imaged tumours. (H) Convolution between (D) and (G). (I) Tensor reconstruction of convolved (pre-processed) image of (H). (J) Tumour segment (classification) in (I) incorporating FCM and the proposed multi-channel reconstruction. (K) Tumour segment in (H) using traditional FCM. (The tumour illustrated is a ductal carcinoma in situ.)

Fig 5 depicts the reconstructed images after the application of pre-processing operations on the DCE-MRI datasets. The tumour to be identified is described as an in situ ductal carcinoma. Fig 5(A) illustrates a single layer for the baseline images, and this corresponds to an MR image acquired before the contrast enhancing agent is injected. Fig 5(B) relates to the post contrast enhanced MRI for the same layer at the fourth time frame. (C) Depicts subtraction of (A) from (B). (D) Shows the result from the application of FCM clustering to achieve initial binarization. (E) Shows the application of the morphological dilation operation on the image depicted in (D); (F) Depicts the imaged breast skin region that needs to be extracted. (G) Shows the result from the subtraction of (F) from (E) in order to achieve a 3D image mask for the selection of voxels that are associated with tumours. (H) Shows a convolution between (C) and (G). (I) Shows the tensor reconstruction of the convolved (pre-processed) image of (H). (J) Shows segmentation (classification) of breast tumours in the reconstructed MRI volumes shown in (I) combining with FCM (hybrid segmentation). (K) Shows standard segmentation (classification) of breast tumours in pre-processed MRI volumes with original intensity in (H) using conventional FCM.

### Evaluation of multi-channel reconstruction of tumours

In order to test further the validity of the proposed methodology within the framework of the multi-channel reconstruction of tumours described in the previous sections, 4D sequences for eleven different test cases are considered. The extracted tumour components with the largest enhancement factor are finally clustered and segmented. For visualization purposes, we illustrate the resultant analysis by showing a single layer of the recovered 3D image. This is contrasted with the corresponding layer of FCM classified enhancement-scaled image so that differences between the hybrid classification algorithm and the traditional FCM approach can be established.

Objective image reconstruction from high dimensional datasets is of fundamental importance across all image analysis tasks. Performance evaluation is a challenging task due to the complexity of the associated data structures, the large variety of processing algorithms that need to be considered as well as the lack of a clearly defined and documented ground truth which makes existing algorithms difficult to evaluate. In order to partly address this issue, in this paper, new self-referencing global image quality analysis metrics are proposed in order to circumvent current problems with a lack of a universally accepted reference ground truth. In order to perform a quantitative analysis of the segmented tumour volume images using the proposed hybrid classification method, we compare our results with those obtained using the traditional FCM method as applied to each dynamic volume MR image.

A value of 1 is set to denote the classified tumour regions as obtained using the traditional FCM, and a value of 2 to denote a tumour region recovered using the proposed hybrid method. The overlapping volume regions in the images as associated with both classification methods are labelled as 3. The number of voxels labelled with values 2 and 3 therefore relate to voxels in a tumour region as recovered using the proposed hybrid classified method.

The approach consists of four quality metrics: (i) non-covered to reconstructed ratio, (ii) overlapped to reconstructed ratio, (iii) difference (DIF) to reconstruction ratio, and (iv) noise to reconstruction ratio (NOI/REC) & noise to ES ratio (NOI/ESI). All of them are designed to assess the level of distortion in the reconstructed images. Results associated with the evaluation of these metrics in de-embedding different types of tumours are further discussed in a subsequent paragraph.

For the first two quality matrices, intensity values associated with voxels in locations that are disconnected from the main tumour regions are set to zero, this enables a comparative analysis. The aim is to establish the degree of spatial correlation in intensity values of tumorous regions across images acquired consecutively in time. Such information can elucidate the presence of artefacts in the reconstructed tumours and enable the identification of possible shape variation due to distortions from the reconstruction process.

**Fig 6:**
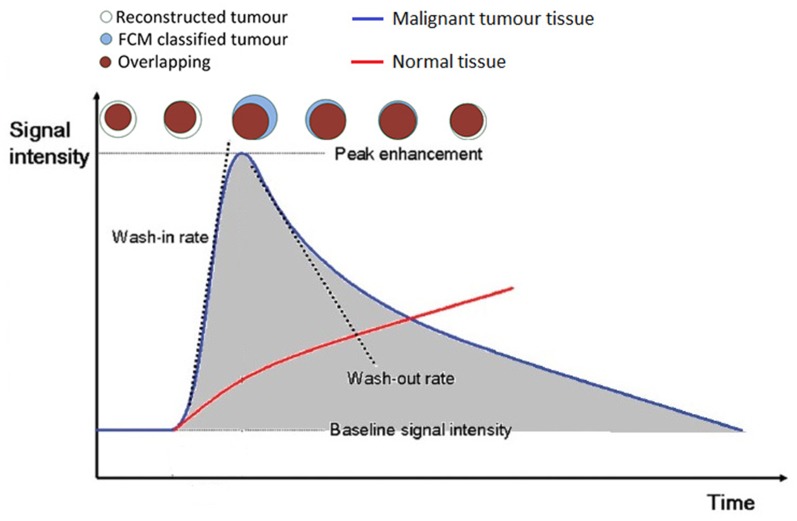
Illustration of the basic principle of the proposed image quality matrix. The top of the figure shows the simulation of a sequence of round shaped tumours detected at different time frames. Regions in blue colour indicate tumour segments identified through the use of FCM, the white regions indicate identified tumour regions using the proposed algorithm; red colour indicate overlapping tumour regions as identified through the use of both algorithms. Typical changes of DCE-MRI signal intensity as a function of time as observed across all images for normal (red) and malignant tumour tissue (blue) are also shown. Wash-in and wash-out rates represent the velocity of enhancement and velocity of loss of enhancement.

Slight changes in signal intensity after the injection of contrast agent can cause significant change in the resulting images of tumours. This is illustrated in Fig 6, where the brown coloured round regions indicate overlapping voxels between the reconstructed tumour segment and FCM ES tumours; the first pattern indicates the difference between the two classified tumour images as acquired at the first time frame after the injection of the contrast agent.

Generally, reconstructed tumours show a variation in size which is well correlated with the intensity curve shown in Fig 6. According to the blue coded signal intensity curve (quick wash in and wash out), for images acquired in the first two time frames, reconstructed tumours show a relatively smaller size than those reconstructed from images at time frames 3 and 4. For images acquired in frames 5 and 6, reconstructed tumours show a relatively larger size than those ES images at time frames 3 and 4. Overall, as shown in subsequent figures, the difference between the time series of enhanced scaled (ES) tumour segments and the reconstructed tumour segment should well reflect the size change of the ES tumour images against time. When there exists good overlap (brown coloured regions) between the reconstruction and ES images, white coloured regions indicate larger size of reconstructed tumours than the ES image at the specific time frame, and blue coloured regions indicate larger size of the ES images at the specific time frame than the reconstructed tumour.

(i) In order to find the voxels that are involved in the FCM based classification of tumour region but non-covered by the reconstructed image segmentation, we define a non-covered to reconstructed ratio (NcReR). We also define NCD as the number of non-covered voxels by the reconstructed tumour segment. REC refers to the number of voxels in the reconstructed tumour segment, so that: $\text{NcReR}=\frac{\text{NCD}}{\text{REC}}$.

(ii) In order to find the overlapping between the reconstructed tumour voxels and the FCM ES image, we define an overlapped to reconstructed ratio (OvReR). Note that OVL is the number of overlapped voxels counted from the reconstructed tumour region with the proposed algorithm and the pixels associated with the FCM ES image. OVL is defined as: $\text{OvReR}=\frac{\text{OVL}}{\text{REC}}$. This quality metric essentially shows the degree of similarity in the two reconstruction methods.

(iii) A difference (DIF) to reconstruction ratio (DiReR) metric is also defined. Note that DIF is composed of two types of voxels: those associated with the original enhanced images but not from the reconstructed tumour images (TuRI), and those tumour voxels associated with reconstructed tumour images but not from original enhanced images (TuOI). Based on the above definition, DiReR is defined as: $\text{DiReR}=\frac{\text{DIF}}{\text{REC}}$, where DIF = TuRI + TuOI, TuRI ∩ TuOI = 0. The purpose of this index is to elucidate differences in tumour reconstruction from both the FCM ES algorithm as well as the newly proposed algorithm when these are applied to images obtained at different time frames after contrast agent injection.

(iv) Spatially isolated voxels (not connected to the main tumour) that are found in neighbouring regions near the main tumour are also accounted for, these are identified as noisy (NOI) voxels (either from ES image (ESI) or from the reconstructed image (REC)). The following ratios can then be defined: noise to reconstruction ratio (NoReR) and noise to ES ratio (NoEsR). These two ratios are defined as: $\text{NoReR}=\frac{\text{NOI}}{\text{REC}}$, and $\text{NoEsR}=\frac{\text{NOI}}{\text{ESI}}$.

The values of OVL/REC and DIF/REC are shown in Fig 17. In order to further illustrate more clearly the changes associated with each image, an offset is applied on each one of the calculated ratios mentioned above. The use of blue colour indicates an averaged intensity in the tumour region of the pre-processed volume images at six different time frames. The green dash and red dash dot curves denote the OVL/REC and DIF/REC ratios for the six time slices, respectively.

#### Removal of intensity inconsistencies through multi-channel reconstruction

**Fig 7:**
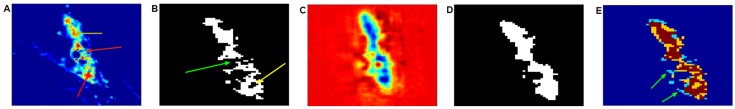
Tumour segment reconstruction from multiple channels. (A) Illustration of intensity variation for breast tumour tissue images; yellow arrows indicate a high intensity and red arrows low intensity. (B) Illustration of FCM based segmentation on (A) with inhomogeneous boundaries; yellow arrows indicate an irregular ring region and a green arrow indicates missing areas. (C) Reconstructed volume image from multiple channels. (D) Tumour segment after reconstruction. (E) Overlapped images (brown) between original tumour segment (blue) and reconstructed tumour segment (yellow). The green arrow indicates the fuzzy edges associated with the original image. The imaged tumour corresponds to an in situ ductal carcinoma.

Changes in intensity of imaged tumours in MRIs are common in clinical practice and as a consequence this leads to an inherent difficulty in the segmentation of an object of interest. This variation is mainly attributed to intra-scan intensity inhomogeneities. Susceptibility artefacts in gradient echo images are known to affect frequently the observed intensities, causing significant intra-scan intensity variation [41]. Therefore, although MRI images may appear visually uniform, the intra-scan inhomogeneities often scramble intensity-based segmentation. A typical example of such an intra-scan intensity inconsistency for a tumorous breast tissue is illustrated in Fig 7(A), which depicts a ductal carcinoma (malignant tumour) in situ. Although the parts depicted by the arrows show the same anatomical structure taken from the same tumour region, the intensity values are different (yellow arrows denote higher intensity than red arrows). After conducting intensity based segmentation, i.e. FCM, as illustrated in Fig 7(B), a region with low intensity in the shape of an irregular ring with a hole inside is reconstructed, in some cases the reconstruction process may also produce disconnected regions separated by a gap.

The resulting volume image as reconstructed from multiple channels using the new procedure is illustrated in Fig 7(C). A greatly improved intensity consistency with the tumorous regions colour-coded in blue clearly separated from the red background region can be seen. Fig 7(D) illustrates the resultant segment of the tumour after reconstruction. When this is compared with Fig 7(B), where the whole region of tumour shows intensity inhomogeneity as well as missed and spurious edges, the resulting segmentation through the multi-channel reconstruction shows homogeneous boundaries for the tumours; in addition, the entire tumour shape and tumour position are clearly retrieved. This can be further validated by observing the overlapping images, shown in Fig 7(E), where the yellow region consists of voxels mainly from the reconstructed tumour segment used to fill in the missing voxels from the conventional FCM classified image. As a result, the reconstruction enables to correctly locate tumour boundaries while eliminating spurious detection of tumours that can occur through the standard FCM reconstruction process. The proposed hybrid classification leads to a better segmentation of the enhanced patterns compared to the FCM based reconstructed segments which show heterogeneous internal enhancement patterns containing artefacts.

#### Suppression of background voxels through multi-channel reconstruction

**Fig 8:**
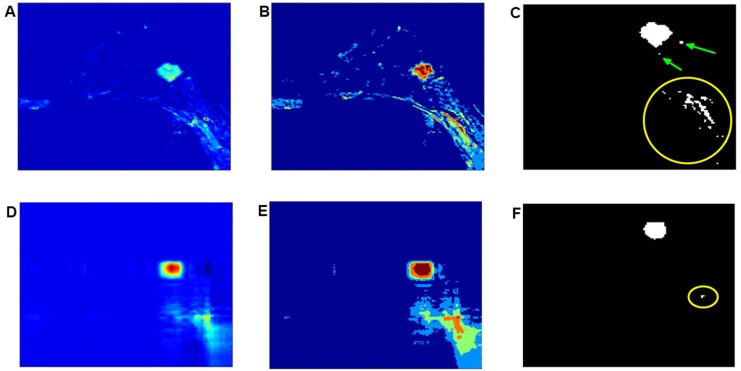
Investigating the effect of spatial intensity inhomogeneities to the proposed classification and FCM. (A) Illustration of the pre-processed images before reconstruction of a granular cell tumour. (B) Colour coded images after application of the FCM algorithm on (A). (C) Magnification of the extracted tumour region shown in (B). The yellow circle and green arrows indicate a misclassified tumour region. (D) Reconstructed volume image from multiple channels. (E) Illustration of the classified image using the proposed hybrid algorithm. (F) Extracted tumour region according to (E). The yellow circle indicates that fatty tissue regions that are misclassified as tumour regions have been shrunk to a very small region depicted as a single dot. This region is small enough to be ignored.

Intensity-based classification of MR images has proven to be the Achilles heel to all automated segmentation methods. For example, when differentiating between tumorous from healthy breast tissue, the inter-scan or spatial intensity variations often originate from the presence of inhomogeneous magnetic field gradients in the MRI equipment during the image acquisition process. These field variations are often of sufficient magnitude to cause an ambiguity in reconstructed tissue boundaries across different tissue classes to overlap, thereby undermining the fidelity associated with such intensity-based classification. An example of such spatial intensity inhomogeneities is illustrated in Fig 8(A). In this figure, the spatial intensities between background and tumour regions are relatively uniform. It is, therefore, difficult to recognize the tumour region from background images on the basis of a variation in intensity [42]. This is further investigated by comparing, the results obtained using the proposed hybrid classification and standard FCM classification algorithms. FCM classification is applied on each of the originally dynamic enhanced images.

Fig 8(A) illustrates the original enhanced image associated with a scan at the second time frame. After applying FCM, the red and brown regions shown in Fig 8(B), correspond largely to the tumour region. The blue and green regions in Fig 8(B), correspond mainly to the background. The extracted tumour regions, as shown in Fig 8(C), also include imaged fatty tissues, these are indicated by a yellow circle. The two green arrows denote misclassified tumour regions. The proposed multi-channel reconstruction addresses well the problem of removing misclassified tumour voxels because it consistently produces images showing a consistent depression of the intensity associated with all fatty tissue. Compared to the image in Fig 8(A), where there is no obvious variation in intensity between the tumour region and the background, the multi-channel reconstruction shown in Fig 8(D), attributes most of the image intensity to the local tumour region and better differentiates tumorous from fatty tissue and background, this is as indicated by a yellow circle at the bottom right section of the recovered image, shown in Fig 8(F). This result can be further visualized based on the proposed hybrid classification assuming 5 classes, as illustrated in Fig 8(E). It can be seen that the tumour region is mainly colour coded in brown whereas background tissue is colour coded in red, green and blue. The extracted tumour regions including the imaged fatty tissue are shown in Fig 8(F). The FCM classified imaged fatty tissues indicated by a large yellow circle shown in Fig 8(C) have shrunk to a single voxel as indicated by the small yellow circle shown in the recovered image. It should be highlighted that in Fig 7(C), the size of the region associated with noise pixels is nearly comparable with the size of the region associated with the tumour, which leads to difficulty in distinguishing between tumorous and healthy tissues. The proposed hybrid classification shown in Fig 7(D) makes easier to identify different tissue types. Reconstruction based on information from the first channel only, recovers well tumorous voxels from background, and this recovery is also correlated with an overall depression in the intensity of the imaged background tissue.

#### Increased image contrast between tumours and background through multi-channel reconstruction

**Fig 9:**
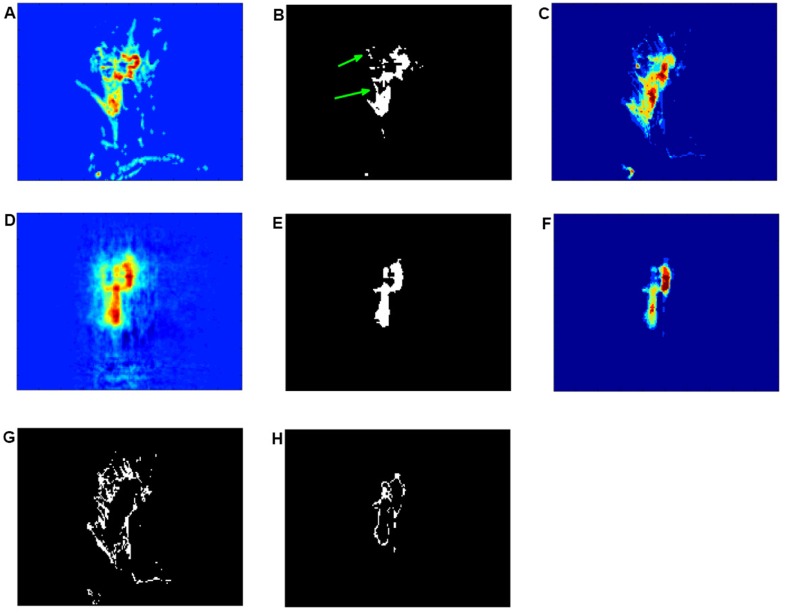
Assessment of an incremental change in intensity contrast between tumours and background through multi-channel reconstruction. (A) Pre-processed images of invasive ductal carcinoma before reconstruction. (B) Illustration of the extracted tumorous regions after the application of FCM classification. The green arrows indicate misclassified healthy tissue regions as tumorous regions. (C) Superposition of images after conducting FCM classification for the identified tumour region. (D) Illustration of tumour reconstruction. (E) Resultant classified tumours through the proposed hybrid approach. (F) Superposition images after conducting the proposed hybrid classification. (G) Magnification of the extracted fuzzy edges of the FCM classified tumour segments shown in (C). (H) Detail of the smooth reconstructed edge shown in (F).

Due to a different intensity distribution associated with different types of tissues, in theory, the background voxels should be more easily separable from tumorous tissue voxels. Frequently, however, interscan intensity inhomogeneities lead to an erroneous depiction of background fatty tissue, and tumour tissue can appear co-located across different parts of the image, as shown in Fig 9(A). As a consequence, in certain cases it can become difficult to define clear boundaries. This is further illustrated in Fig 9(B), where a single layer associated with the second sequential FCM segment of the enhanced imaged tumour is displayed, and Fig 9(C), where sixty layers of identified tumours are superposed after FCM classification. The regions coded in light blue (brighter than background blue) illustrated in Fig 9(C) correspond to imaged fatty tissue voxels (G). This region shows several large fuzzy edges, which implies that many regions of fatty tissue have been misclassified as tumorous tissue. In this case, a clear boundary between tumorous and background tissue needs to be defined. This can be achieved through step-by-step systematic increases in intensity contrast. The first channel reconstruction of imaged tumours, as shown in Fig 9(D), addresses well this problem. Compared with Fig 9(B), where some joined heathy tissues are clearly visible, as indicated by green arrows, Fig 9(E) preserves the whole spatial structure of tumours and removes the fatty tissue related background region that has been misclassified as tumorous, this is further illustrated in Fig 9(B). Sixty layers of identified tumours in the reconstructed image are superposed and shown in Fig 9(F). After classification using the newly proposed hybrid approach, regions denoted by light-blue voxels can be extracted, as illustrated in Fig 9(H). The classified voxels form a clear edge region around the tumours, and remove all fuzzy edges as shown in Fig 9(G). The proposed multi-channel reconstruction therefore enables us to achieve uniformly enhanced intensity distributions for all image regions associated with the tumours. Furthermore, increased image contrast between tumours and background is also achieved.

#### Limitations associated with multi-channel reconstruction

**Fig 10:**
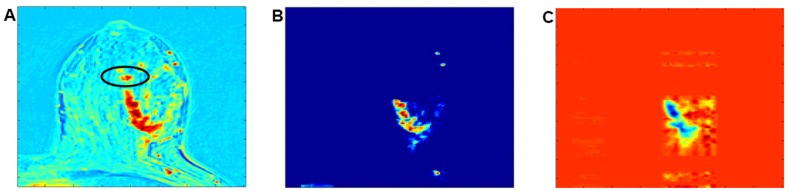
Illustration of a reconstruction procedure. (A) Illustration of a single layer of the originally enhanced image acquired at the fourth time frame (B) Illustration of the pre-processed image before reconstruction. (C) Reconstructed volume image using information from multiple channels (The image is from an invasive ductal carcinoma).

There are two reconstruction problems that have been encountered in the current study. In situations where multi-channel reconstruction is performed on breast DCE-MRIs on very large tumours, e.g., in cases of images depicting an invasive ductal carcinoma as illustrated in Fig 10(A), it is often difficult to reconstruct the entire tumour. Since reconstruction is performed on the basis of information acquired from only six time frames, for large sized tumours, a significant number of voxels can be redundant whereas the number of time frames is rather limited; this causes difficulty to achieve an effective reconstruction. Fig 10(B) depicts such situation, where the black circled part of the tumour in Fig 10(A) has been removed in order to reconstruct the remaining part of the tumour image more accurately. This is illustrated in Fig 10(C), where most of the tumour spatial structure can be well reconstructed, with the exception of the black circled region in (A).

**Fig 11:**
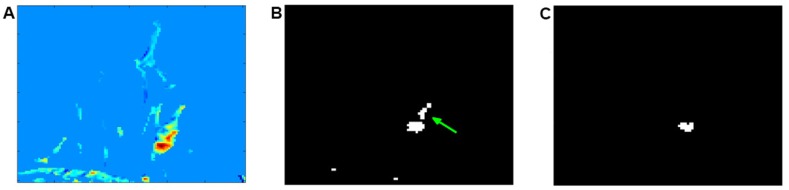
Illustration of a reconstruction procedure. (A) Single layer of the originally enhanced (ES) image acquired at the fourth time slice. (B) Single layer of the extracted tumours from the pre-processed image before reconstruction. (C) Single layer of the extracted tumours using the proposed hybrid classification (The images correspond to a malignant mucinous carcinoma).

It was also found that when performing multi-channel reconstruction of a mucinous carcinoma, as shown in Fig 11(A), not all the voxels from the identified tumour region can be fully reconstructed. This is further illustrated in Fig 11(B), where the part indicated by a green arrow could not be fully reconstructed; in that case, the resultant partial reconstruction is shown in Fig 11(C).

#### Evaluation of lesion reconstruction using predefined qualitative metrics

One of the aims of the validation procedure is to investigate the ability of the proposed reconstruction method in achieving a uniform distribution from tumour voxels. A clinical requirement for reconstruction is that the continuity of the entire tumour region should not be broken through the reconstruction process, thus ensuring no artefacts are incorporated through the reconstruction process. This way, an entire tumour region after reconstruction should form a continuous surface. Disconnected tumour voxels are thus classified as noise/artefact voxels. However, due to tissue heterogeneity present on tumours, the intensities of imaged tumour are not consistent, furthermore, not all the tumour voxels are enhanced to the same extent. Non-enhanced tumour voxels can occasionally be misclassified as imaged background fatty tissue, and this can result in some tumour voxels becoming disconnected. To evaluate the ability of the current reconstruction methodology to convert the non-enhanced intensity tumour voxels to enhanced intensity tumour voxels, we estimate the number of tumour voxels that are not connected to the main classified tumour region (where most classified tumour voxels can be found to have a spatial connection with each other). For simplicity, we view these non-connected tumour voxels as noise-labelled voxels. Whenever the proposed reconstruction process enhances some tumour voxels, some non-connected tumour voxels become re-connected. As a consequence, the non-connected tumour voxels that have been viewed as noise voxels change in size. A metric is thus devised to assess the fidelity of the reconstruction process. In this metric, a smaller number of noise voxels implies a more accurate reconstruction.

**Fig 12:**
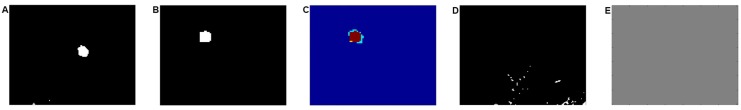
Illustration of an invasive ductal carcinoma (case I). (A)-(E) Segmented tumours using FCMs and the proposed hybrid classification, overlapping images, and depiction of noisy voxels from imaged fatty tissue, using both classification methods; absence of noisy voxels in E relate to results from the proposed algorithm.

**Fig 13:**
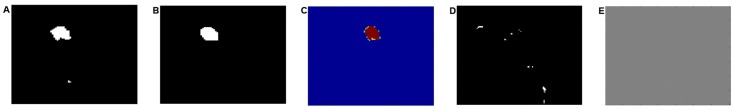
Illustration of a fibroadenoma (case II). (A)-(E) Segmented tumours using FCMs and the proposed hybrid classification, overlapping images, and depiction of noisy voxels from imaged fatty tissue, using both classification methods; absence of noisy voxels in E relate to results from the proposed algorithm.

**Fig 14:**
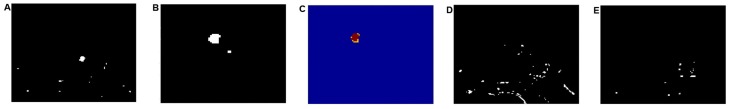
Illustration of focal lobular neoplasia (case III). (A)-(E) Segmented tumours using FCMs and the proposed hybrid classification, overlapping images, and depiction of noisy voxels from imaged fatty tissue, using both classification methods; the fewer noisy voxels in E relate to results from the proposed algorithm.

**Fig 15:**
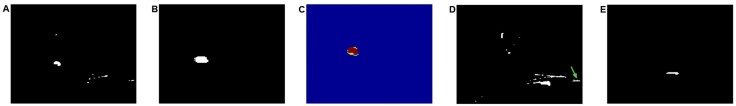
Illustration of a tubular adenoma (case IV). (A)-(E) Segmented tumours using FCMs and the proposed hybrid classification, overlapping images, and depiction of noisy voxels from imaged fatty tissue, using both classification methods. The green arrow seen in (D) indicates one of the very small regions of noise that is related to the noise region shown magnified in (E).

**Fig 16:**
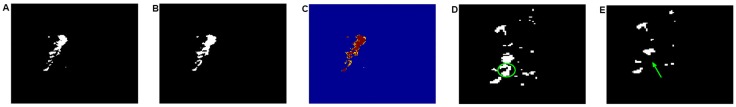
Illustration of an invasive ductal carcinoma (case V). (A)-(E) Segmented tumours using FCMs and the proposed hybrid classification, overlapping images, and depiction of noisy voxels from imaged fatty tissue, using both classification methods. The disconnected tumour voxels seen in (D), indicated by a green circle, are identified as a connected tumour component attached to the major tumour region, the reconstruction is performed using the proposed hybrid classification, these can also be seen in (E) next to the green arrow.

This metric is used to assess the reconstruction fidelity of five sets of DCE-MRIs, these images are associated with four different types of tumours: invasive ductal carcinoma (malignant), fibroadenoma (benign), focal lobular neoplasia (benign), tubular adenoma (benign), and invasive ductal carcinoma (malignant). For simplicity, we shall refer to these as Cases I, II, III, IV and V respectively. In order to capture the spatial and temporal aspects of the original images, from Figs 12 to 16, we compare the resulting segmentation after reconstruction through the proposed hybrid classification and standard FCMs.

The reconstructed images for Case I are illustrated in Fig 12. Fig 12(A) relates to a single layer of FCM classified tumours on enhancement-scaled images (ES). Fig 12(B) illustrates a single layer of the reconstructed tumour segment selected from the first channel. A single layer of overlapped images between the FCM tumour segments of ES and the tumour segment reconstructed by the new hybrid method is illustrated in Fig 12(C), with light blue labelled regions indicating the voxels from the ES related segments via as reconstructed by FCMs, the yellow labelled regions indicating the voxels from the proposed classification and the brown labelled regions indicating the overlapped voxels. The size of the hybrid classified tumour region is slightly smaller than the extracted tumour region of the FCM classed ES image. Fig 12(D) shows the superposed FCM classified tumour voxels that are disconnected. It is worth contrasting these with the reconstruction shown in Fig 12(E) where the number of noise voxels in relation to classified tumour using the proposed approach is zero, whereas the number of noisy voxels in Fig 12(D) is relatively large.

The same conclusion can be obtained when comparing Fig 13(A) and 13(B) which show the extracted tumour segments using FCMs and our the new approach. The relevant overlapped image in Fig 13(C) illustrates that the shape and size of the reconstructed tumour segments using both methods are similar but the reconstruction using the hybrid algorithm has no noisy voxels associated with the fatty tissue, in contrast the number of voxles in the FCM classified ES image is large.

Case III shown in Fig 14(A) depicts FCM extracted tumour segmentation also containing several noisy voxels. Again, the hybrid algorithm shows a very small region of noise. Fig 14(B) provides a magnification of the segmented tumour where the size of noisy regions is so small that it can be ignored for most practical purposes. Fig 14(C), also shows good overlap of the segmented tumours between the two algorithms. The superposed imaged noise after using FCM classification can be seen in Fig 14(D). Fig 14(E) shows very small number of reconstructed noisy voxels compared with Fig 14(D).

Case IV is illustrated in Fig 15, where Fig 15(A)–15(C) are the tumour segments reconstructed using FCMs and our the hybrid approach, and these are images with good overlap overall. The accumulated noise voxels using FCMs and the proposed algorithm are illustrated in Fig 15(D) and 15(E). The noise in subfig. (E) is shrunk into a very small region, (indicated by the green arrow in subfig. (D)).

In Case V, shown in Fig 16(A)–16(C) there is also good overlap in the reconstruction process with the hybrid algorithm again performing better than FCM. This can also be seen in Fig 16(D) and 16(E) where the disconnected tumour voxels, (indicated by green circle), have been identified as a connected tumour component attached to the major tumour region using the hybrid classification. It can also be seen (subfig. (E)), that there are no voxels in the corresponding region, as indicated by a green arrow. Some of the segmented images are magnified to improve visualization.

In Figs 12 to 16, we depict the following five specific ratios: Non-covered to reconstructed ratio (NCD/REC); overlapped to reconstructed ratio (OLP/REC); difference to reconstruction ratio (DIF/REC); noise to FCM classified ES ratio (NOI/ESI); noise to reconstruction ratio (NOI/REC), the values if these are listed in Table 1.

**Table 1:** Comparison of qualitative tumour segment reconstruction ratios: (i) NCD/REC (NcReR), (ii) overlapped voxels (OvReR), (iii) DIF/REC (DiReR) and (v) NOI/REC (NOI/ESI) (NoEsR/NoReR) for five cases assuming FCM classification of enhanced scaled (ES) images observed at six consecutive time frames and the proposed hybrid tumour classification using one channel.

Case	Image	NcReR	OvReR	DiReR	NoESI/NoReR
I	1st enhanced	59.59	**60.87**	**98.72**	**521.44**
	2nd enhanced	**19.36**	93.09	56.27	64.99
	3rd enhanced	32.23	89.77	**42.46**	**27.88**
	4th enhanced	53.71	96.42	57.29	44.12
	5th enhanced	**67.52**	**98.72**	68.80	45.23
	6th enhanced	67.26	97.70	69.57	51.97
	Hybrid classification				***0***
II	1st enhanced	**12.42**	**88.63**	23.79	**9.07**
	2nd enhanced	14.36	92.60	**21.76**	19.28
	3rd enhanced	16.74	94.71	22.03	18.18
	4th enhanced	17.18	94.63	22.56	18.05
	5th enhanced	**24.58**	97.36	**27.22**	**20.01**
	6th enhanced	21.32	**97.18**	24.14	13.53
	Hybrid classification				***0***
III	1st enhanced	**0**	**0**	**100**	**Inf**
	2nd enhanced	**0**	19.23	80.77	1460
	3rd enhanced	0.55	39.01	61.54	323.61
	4th enhanced	1.65	64.84	36.81	292.56
	5th enhanced	**7.14**	**85.16**	**21.98**	288.69
	6th enhanced	6.59	75.82	30.77	**142.00**
	Hybrid classification				***42.86***
IV	1st enhanced	**13.01**	79.93	**33.09**	**208.00**
	2nd enhanced	**4.46**	**72.49**	31.97	166.18
	3rd enhanced	6.69	79.93	26.77	172.53
	4th enhanced	10.78	**85.87**	24.91	191.54
	5th enhanced	9.67	**85.87**	**23.79**	**161.09**
	6th enhanced	9.67	84.01	25.65	167.06
	Hybrid classification				***13.01***
V	1st enhanced	**1.97**	**64.67**	**37.30**	16.61
	2nd enhanced	2.80	79.44	23.35	17.42
	3rd enhanced	3.22	83.03	20.20	**19.44**
	4th enhanced	4.23	85.87	**18.36**	12.97
	5th enhanced	11.67	**92.19**	19.48	15.86
	6th enhanced	**15.16**	92.05	23.11	**7.69**
	Hybrid classification				***7.77***

**Fig 17:**
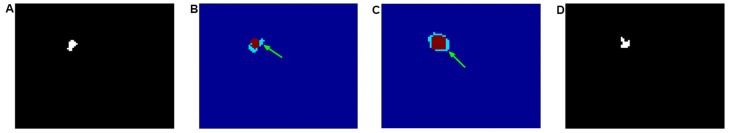
Plots showing the correlation between of average intensity and two qualitative metrics for images obtained at different time frames. (A)-(E): Illustration of the DIF/REC ratio (red dash dot curve), OVL/REC ratio (green dash curve) and averaged intensity (blue curve) in the tumour region of interest across for the five tumour cases considered. The *x*-axis labels the six different time steps, and the *y*-axis labels the scaled value of each ratio and intensity.

We also plot the overlapped to reconstructed ratio (OLP/REC) and the difference to reconstruction ratio (DIF/REC), which is illustrated in Fig 17. We introduce different offset, in order that the ratios can be compared with the averaged intensity in classified tumour regions. The blue solid line shows the average intensity at each time frame; this is calculated on the basis of intensities of ES voxels within the FCM classified imaged tumour regions. The red dash dot line shows the difference to reconstruction ratio DIF/REC at six different time frames. These lines show that there is some correlation between the average intensity, and DIF/REC with a reduction of the average intensity associated with an increased DIF/REC ratio. The green dash lines show the OLP/REC ratio as calculated at the six different time slices. When the OLP/REC ratio is increased, the DIF/REC ratio is reduced; for reduced OLP/REC ratio, there is an increase in the DIF/REC ratio. This correlation is not, however, always consistent, for example, in the case shown in Fig 17(B), the above correlation is not as good.

**Fig 18:**
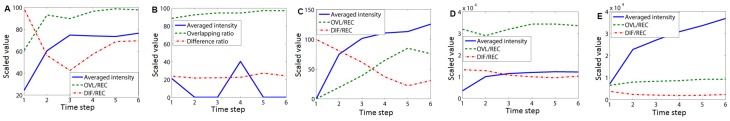
Overlapped volume image based on hybrid classification of imaged tumours and the FCM classified ES images associated with the fifth time frame for case I. (A) Overlapped tumour segments at the first imaging layer. (B) and (C) Overlapped tumour segments randomly selected from two imaging layers. (D) Overlapped tumour segments reconstructed from the last imaging layer.

Table 1 shows the NCD/REC ratios for the case of an invasive ductal carcinoma (case I). It can be seen that this ratio is highest at the 5th time frame of the FCM classified ES image, and lowest at the second time frame, while nearly all the OLP/REC ratios are higher than 90% with higher DIF/REC ratios than 42.46%. It should also be noted that the NOI/ESI ratio is very high, up to 521.44% according to the FCM classified ES images taken at different time frames, while the NOI/REC ratio according to the reconstructed image is zero. This indicates the superior fidelity of the reconstructed tumour segment achieved through the proposed hybrid classifier method. Unfortunately, for case I, a reasonably large DIF/REC ratio is obtained, which is undesirable. This, however is not the case for all the other cases of tumour types. By comparing the overlapped images obtained at the fifth time frame, between each one of the FCM based ES images and the hybrid classifier images, illustrated in Fig 18, it can be seen that the first and last layers of the 3D tumour region are missed after using the proposed approach, this is shown in more detail in subfig. (A) and subfig. (D). The segmentation of multi-channel reconstruction shows slight shrinkage along the edge of the imaged tumour compared with the FCM classification, as shown in subfig. (B) and subfig. (C).

Table 1 shows the NCD/REC ratios for the case of a fibroadenoma (case II). It can be seen that this ratio is less than 24.58%, indicating that the number of non-covered voxels by the proposed hybrid classifier compared with FCM classification of ES tumours is low. Nearly the same size and shape in the detected tumours is obtained using these two methods. Nearly all of the OLP/REC ratios are over 92% and the DIF/REC ratios are less than 27.22%, indicating identical tumour segments were identified using these two classification methods. The NOI/ESI ratios are higher than 9.07%, while the proposed algorithm shows a NOI/REC ratio of zero, which indicates a good reconstruction quality compared with the FCM classified ES images. This showcases the merits of the hybrid algorithm proposed.

Finally, Table 1 shows the NCD/REC ratios for the case of a tubular adenoma (case IV) and an invasive ductal carcinoma (case V). For both these cases, the NCD/REC ratios are very low, (mostly below 10%), and the OLP/REC ratios are high, indicating small changes in the size and shape of the reconstructed tumours and FCM classified tumours from ES images. Most of the DIF/REC ratios for Case IV are lower than 26.77% but with greatly improved NOI/REC ratio of 13.01% (that is over 12 times lower than the NOI/ESI ratio, further confirming the advantages of the proposed hybrid algorithm). For case V, the ratio of DIF/REC is between 37.30% and 18.36%. This is acceptable considering that the low contrast in intensity of the ES image can result in some details being missed in the FCM classified tumour region of interest. With increased contrast, the DIF/REC ratio is further reduced. As the current algorithm allows converting the non-enhanced tumour voxels to enhanced tumours voxels, this leads to more tumour voxels being connected, depicting an entire tumour region with consistent image intensity. In order to further identify how much missing information can be picked up after the proposed classification technique, one can examine both the NOI/REC and NOI/ESI ratios. The NOI/REC ratio is low compared to nearly all the observed NOI/ESI ratios, indicating the algorithm’s ability to improve the homogeneity in the reconstructed tumours even though there are often several heterogeneous features on the intensity distribution in the original measurements.

## Conclusion

Current DCE-MRI diagnosis on disease proliferation is not sufficiently accurate when applied to the early detection of tumours because of a lack of information relating spatial & and temporal features. A new hybrid methodology has been proposed for the extraction of spatiotemporal features paving the way for automated tissue diagnosis. The new approach performs transformations in both spatial and temporal domains. Spatial and temporal features of DCE-MRI are reconstructed through a multidimensional unified analysis of MRI data under a tensor algebra framework. One of the advantages of the proposed reconstruction is to also incorporate temporal information associated with disease proliferation to achieve spatial and temporal information fusion with decreased number of dimensions at reduced computational overhead. The algorithm uses a high-order singular value decomposition (HOSVD) in the spatial domain incorporating a temporal PCA to effectively extract the dominant modes of temporal variation through a linear combination of basis image time series. During the reconstruction process, covariance information is used to extract voxels associated with the location of the tumour. The multichannel approach provides additional noise suppression originating from fatty tissue. Furthermore, an enhanced intensity contrast is achieved in the reconstructed images. This enables maximum visual separability of image features that are both spatially and temporally related. This leads to better ground truth assessment thus improving fidelity in pattern classification.

As high dimensional data may be sparse, the projection of high dimensional data onto low dimension subspaces enables sparse data to be clustered more efficiently. It was shown that the process of projecting the data to a lower dimension enables the reconstruction of the volume image with a more consistent intensity, converting non-uniformly enhanced ES images to homogeneously enhanced images. There are several advantages of the proposed algorithm. Intensity values in many voxels associated with fatty tissue that are misclassified as belonging to a tumour region using FCMs have been supressed through the proposed multi-channel reconstruction. Even in cases where images depict large sized tumours, the proposed methodology enables the recovery of tumour shape and position information, with consistency in the recovered intensity across the tumour region. For the validation of the proposed algorithm, four different reconstruction quality metrics are defined, these should also find applications in assessing other DCE-MRI tumour reconstruction algorithms.

## Supporting information

S1 Data Multi-channel reconstruction via DCE-MRIs. DCE-MRI Data is used for tumour identification via multi-channel reconstruction.
(RAR)
