# Correction: Tensor based multichannel reconstruction for breast tumours identification from DCE-MRIs

**DOI:** 10.1371/journal.pone.0176133

**Published:** 2017-04-13

**Authors:** X. -X. Yin, S. Hadjiloucas, J. -H. Chen, Y. Zhang, J. -L. Wu, M. -Y. Su

There is an error in affiliation 4 for author J. -H. Chen. Affiliation 4 should be: Department of Radiology, E-Da Hospital and I-Shou University, Kaohsiung, Taiwan.
